# B Lymphocytes Accumulate and Proliferate in Human Skin at Sites of Cutaneous Antigen Challenge

**DOI:** 10.1016/j.jid.2021.06.038

**Published:** 2022-03

**Authors:** Isioma U. Egbuniwe, Robert J. Harris, Mano Nakamura, Frank O. Nestle, Arne N. Akbar, Sophia N. Karagiannis, Katie E. Lacy

**Affiliations:** 1 St. John’s Institute of Dermatology, School of Basic & Medical Biosciences, King’s College London, London, United Kingdom; 2 Translational Medical Sciences Unit, School of Medicine, University of Nottingham Biodiscovery Institute, Nottingham, United Kingdom; 3 Sanofi Immunology and Inflammation Research Therapeutic Area, Cambridge, Massachusetts, USA; 4 Division of Infection and Immunity, University College London, London, United Kingdom; 5 Breast Cancer Now Research Unit, School of Cancer & Pharmaceutical Sciences, King’s College London, Guy’s Cancer Centre, London, United Kingdom

**Keywords:** CLA, cutaneous lymphocyte antigen, VZV, varicella-zoster virus

To the Editor

B cells play important roles in skin diseases (Egbuniwe et al., 2015) and in cutaneous homeostasis (Geherin et al., 2016, 2012; Nihal et al., 2000). Mature class-switched IgG^+^ B cells have been detected in normal human skin (Saul et al., 2016) featuring clonally restricted B-cell receptors, indicating narrow antigenic repertoires (Nihal et al., 2000). However, the involvement of B cells during an antigenic stimulus in human skin remains unexplored. B cells are relatively scarce in normal human skin (Supplementary Figure S1), explaining why past studies have primarily focused on T cells, which constitute the major skin-resident lymphocyte population (Clark et al., 2006b; Jiang et al., 2012; Sanchez Rodriguez et al., 2014).

We investigated the dynamics of B-cell infiltration in the skin after local antigen challenge with varicella-zoster virus (VZV; provided by Prof A.N. Akbar, University College London, UK) and candida (Candin) antigens (Allermed Laboratories, San Diego, CA) by taking skin biopsies from and inducing suction blisters over the site of intradermal injection in human skin in vivo. The induction of skin suction blisters has been reproducibly employed in examining the cellular kinetics of skin-infiltrating immune subsets, including T cells in delayed-type hypersensitivity responses after intradermal injection of recall antigens (Vukmanovic-Stejic et al., 2013); innate lymphoid cells after challenge with house dust mite (Salimi et al., 2013); and different leukocyte subsets (including B cells) during acute inflammation (Akbar et al., 2013; Jenner et al., 2014).

Flow cytometric analysis (FACSCanto II or LSRFortessa Cell Analyser - Becton Dickinson, Franklin Lakes, NJ) showed CD19^+^CD20^+^ B cells in low numbers (mean percentage of total lymphocyte population) in fluid from skin suction blisters induced without antigen challenge (mean = 0.05%, n = 3) (Figure 1a and b). This, in addition to immunofluorescence studies on normal skin (Supplementary Figure S1), confirmed that B cells were scarce under homeostatic conditions in unperturbed skin. After intradermal challenge with VZV antigen, CD20^+^ B cells (percentage of total live lymphocytes) were detected on day 3 (mean = 0.26%, n = 3) and day 7 (mean = 5.07%, n = 3) blisters (Figure 1c). Blister fluid obtained from sites injected with sterile saline (Advanz Pharma, London, UK) contained lower percentages of B cells on both day 3 and day 7 compared with VZV blisters (Figure 1c). A trend toward lower absolute numbers of cells extracted from saline blister fluid (n = 5, mean = 5.1 cells per ml) than that from VZV blister fluid (n = 5, mean = 48.1 cells per ml) was seen, although this did not reach statistical significance.

**Figure 1:**
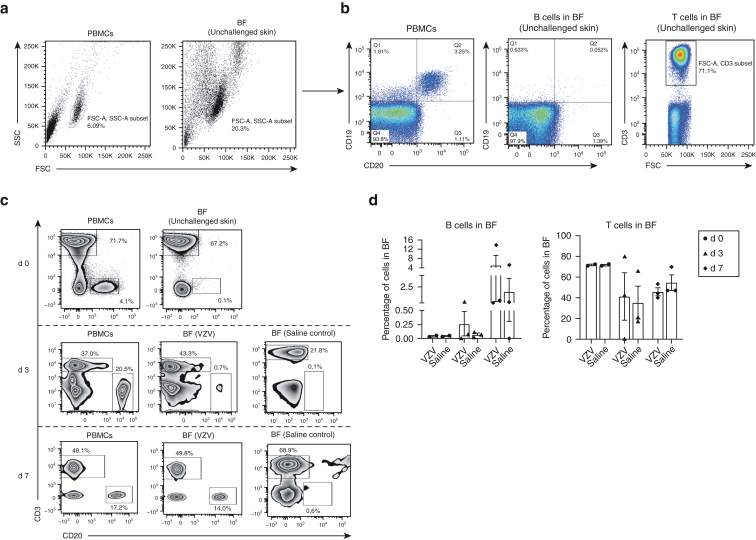

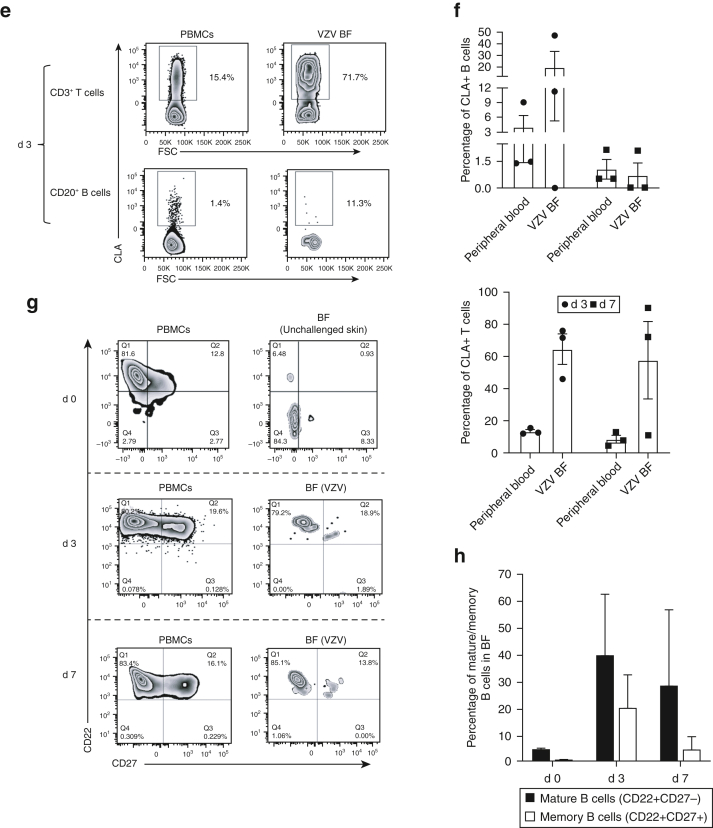

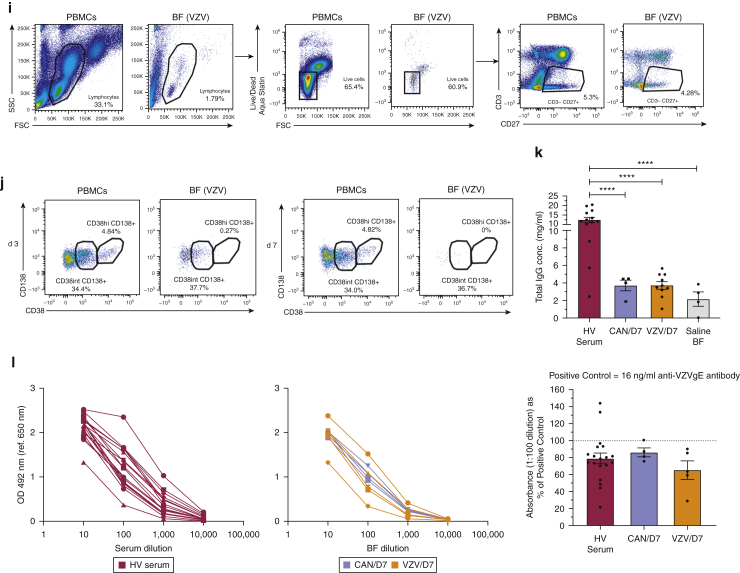
**Characterization of skin-resident B cells with and without VZV antigen challenge.** Suction blisters were induced (without VZV antigen challenge) on the skin of healthy individuals (n = 3), and BF was collected within 24 hours after blister formation for flow cytometric (FACS) analysis. Matched donor PBMCs were also analyzed by FACS. (**a**) Lymphocytes were identified on the basis of SSC and FSC profiles in peripheral blood and skin suction blisters. (**b**) Proportions of CD19^+^CD20^+^ B cells and CD3^+^ T cells were evaluated from the total lymphocyte population within donor PBMCs (n = 3; percentage mean ± SEM; B cells = 3.6 ± 0.3; T cells = 62 ± 19) and unchallenged BF (n = 3; percentage mean ± SEM; B cells = 0.05 ± 0.01; T cells = 53 ± 16). Representative FACS plot shown for an individual donor. (**c**) Donors received intradermal injections of VZV antigen or sterile saline, and blisters were raised over injection sites. Blister aspirates and matched donor PBMCs collected either 3 d (n = 3) or 7 d (n = 3) after injection were analyzed by FACS for B (CD20^+^) and T (CD3^+^) cells. Blisters on d 0 (n = 2) were induced without previous VZV challenge. Representative FACS plot shown for an individual donor. (**d**) Quantification of B and T cells within d 3 (n = 3) and d 7 (n = 3) VZV and saline blister aspirates compared with those within d 0 (n = 2) blisters. (**e**) d 3 blister aspirates and PBMCs analyzed by FACS for expression of CLA (a skin-homing marker) on CD20^+^ B cells and CD3^+^ T cells. Plots represent an individual donor. (**f**) Quantification of CLA^+^ B and T cells within d 3 (n = 3) and d 7 (n = 3) VZV blister aspirates and matched donor PBMCs. (**g–h**) Phenotypic analysis and quantification of mature (CD20^+^CD22^+^) and memory (CD20^+^CD27^+^) B cells within d 0 (n = 2) donor PBMCs and blister aspirates (non-VZV), compared with those within d 3 (n = 3) and d 7 (n = 3) donor PBMCs and VZV aspirates. Representative flow cytometry plots are shown. Kruskal‒Wallis test with Dunn’s multiple comparisons post-test was used for analysis. Error bars represent mean ± SEM. (**i**) Live lymphocytes were identified within peripheral blood and BF on the basis of SSC and FSC profiles and by staining with a live/dead (viability) marker, followed by identification of CD3^–^CD27^+^ lymphocytes. (**j**) Identification of plasma cell (CD38^hi^CD138^+^) and plasmablast (CD38^int^CD138^+^) subsets from within CD3^–^CD27^+^ B cells in peripheral blood and BF. FACS plots are shown for one individual each evaluated on d 3 and d 7. (**k**) Total IgG antibody titres within d 7 BF aspirates from saline- (control, n = 4), VZV- (n = 10), and CAN- (n = 4) challenged skin and from donor sera (n = 13) were analyzed using the ImmunoCAP and Total IgG ELISA assays. Each symbol on the plot represents one individual. ∗∗∗∗*P* < 0.0001. One-way ANOVA with Tukey’s multiple comparison post-test was used for analysis. (**l**) Reactivity of d 7 BF (CAN and VZV) and HV serum IgG to VZV antigen were tested using a modified indirect ELISA assay. Specific signal (optical density) was measured at 1:10, 1:100, 1:1,000, and 1:10,000 dilutions (left hand graphs). Antibody reactivity was then determined as absorbance at 1:100 dilution, relative to a positive control antibody (16 ng/ml anti-VZVgE) for each sample tested (right-hand graph). Error bars represent mean ± SEM. BF, blister fluid; CAN, candida; CLA, cutaneous lymphocyte antigen; conc, concentration; d, day; FSC, forward scatter; FSC-A, forward scatter area; HV, healthy volunteer; K, thousand; SSC, side scatter; SSC-A, side scatter area; VZV, varicella-zoster virus.

Markers of skin-homing B cells remain undefined, although cutaneous lymphocyte antigen (CLA) is expressed on circulating B cells after percutaneous immunization with tetanus toxoid toxin (Kantele et al., 2003). We examined circulating CD20^+^ B cells by flow cytometry for expression of classical T-cell skin-homing markers CLA, CCR4, and CCR10 (Clark et al., 2006a; McCully et al., 2012). The percentages of CLA^+^CD20^+^ B cells were significantly greater than those of CCR4^+^CD20^+^ B cells (no significant differences between CCR10^+^CD20^+^ and CLA^+^CD20^+^ cells) (Supplementary Figure S2). CLA^+^CD20^+^ B cells were identified within VZV blister aspirates on day 3 after challenge (mean = 19.5%, n = 3) and persisted within cutaneous blisters on day 7 after VZV challenge (mean = 0.71%, n = 3). CLA^+^CD3^+^ T cells were present (day 3: mean = 64.6%, day 7: mean = 57.7%, n = 3) (Figure 1e and f) as described (Clark et al., 2006a).

We next investigated antibody production from cutaneous B cells. Flow cytometric analyses revealed populations of mature (CD22^+^CD27^–^) and memory (CD22^+^CD27^+^) B cells within suction blister aspirates on days 3 and 7 after VZV challenge (Figure 1g and h). Steady-state circulating plasma cells lose expression of CD20 but retain high expression of CD27 and CD38, with or without CD138 expression (Caraux et al., 2010). We detected distinct populations of CD38^hi^CD138^+^ in CD3^–^CD27^+^ circulating plasma cells in blood but not in blister fluids of two donors (one assessed on day 3 and one on day 7) after VZV injection (Figure 1i and j). CD38^int^CD138^+^ B cells were also identified within the blister fluid and peripheral blood (day 3 and day 7; Figure 1j), likely representing short-lived plasmablasts known to have antibody-secreting capabilities (Nutt et al., 2015).

VZV-specific IgA and IgG antibodies are produced in response to primary VZV infection, with IgG persisting long term (Arvin, 1996). We detected IgG antibodies (ImmunoCAP assay and Total IgG ELISA; Supplementary Materials and Methods) at higher mean titres within VZV (n = 10, mean = 3.705 mg/ml) and candida (n = 4, mean = 3.688 mg/ml) blister fluids than within saline controls (n = 4, mean = 2.142 mg/ml), although statistical significance was not achieved (Figure 1k). IgG titres within VZV blisters were greater than IgA, IgE, and IgM titres (Supplementary Figure S3). VZV and saline aspirates had lower titres for all antibody subclasses than serum. We also tested the specificity of healthy volunteer serum (n = 19) and day 7 blister fluid (VZV [n = 5] and candida [n = 4]) to VZV-specific IgG antibodies by ELISA. VZV-specific antibodies were found in blister fluid after both VZV and candida antigen challenge in addition to healthy volunteer serum samples, with no significant difference in specific signal among the three groups (Figure 1l; measured at 1:100 dilution and relative to the positive control anti-VZVgE antibody [Merck, Dorset, UK]). These results indicate that it is unlikely that localized antigen-specific antibody production occurs in the skin in response to antigen challenge.

Immunofluorescence studies on skin biopsy sections identified CD20^+^ B cells within dermal perivascular infiltrates 24 hours after VZV and candida skin challenge (Figure 2). We observed the preferential accumulation of CD3^+^ and CD20^+^ immune cells within dermal perivascular areas (Supplementary Figure S4), and we quantified mean CD20^+^ cell density within the three to five most densely populated dermal perivascular areas within skin sections (mean ± SEM). Our analysis revealed significantly increased B-cell numbers from baseline (0.39 ± 0.22; n = 16) to day 1 (n = 5, VZV = 2.56 ± 0.98; n = 3, candida = 5.67 ± 4.70), further increasing through day 3 (n = 5, VZV = 5.60 ± 3.71; n = 6, candida = 8.55 ± 4.35) and day 7 (n = 8, VZV = 10.40 ± 1.82; n = 6, candida = 8.62 ± 2.69) (two sections per donor) (Figure 2a–d). We also found proliferating, Ki67^+^CD20^+^ B-cell infiltrates within VZV- and candida-challenged skin biopsy sections on day 7 (Figure 2f and g).

**Figure 2:**
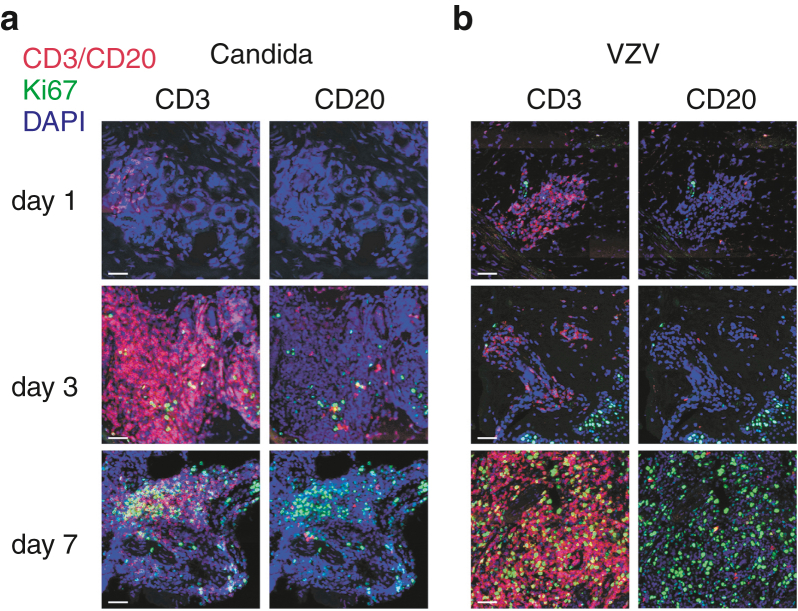

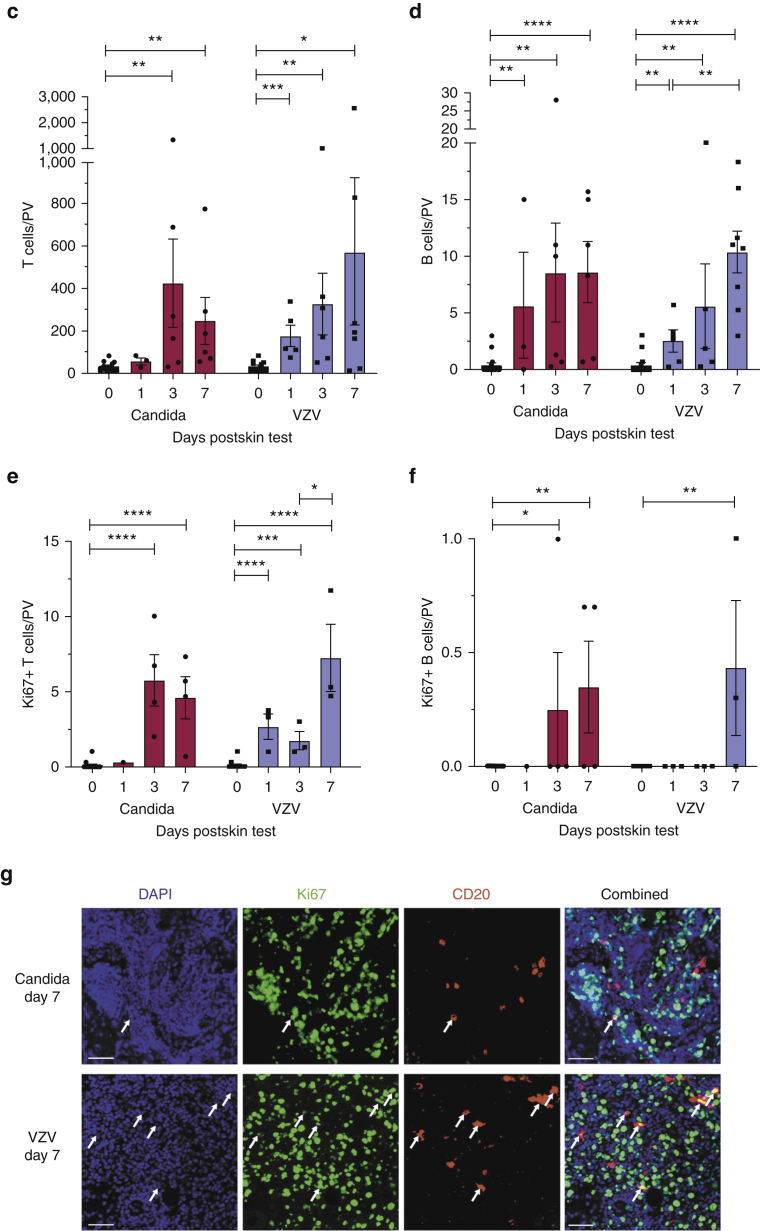
**Human B cells accumulate and proliferate within cutaneous sites of antigen challenge.** (**a, b**) Frozen sections from skin biopsies taken 1, 3, and 7 days after intradermal challenge with (**a**) candida and (**b**) VZV antigens were stained for CD3^+^ T cells (red, left panel), CD20^+^ B cells (red, right panel), and proliferating lymphocytes (Ki67; green) seen within PV areas. All sections were counterstained with DAPI (blue). Original magnification: ×20. Bar = 50 μm. Overall, 3–16 skin biopsies were analyzed per time point (two sections stained per biopsy). Representative images are shown. (**c, d**) Quantification of CD3^+^ T and CD20^+^ B cells within candida and VZV biopsy sections described in **a** and **b**. Day 0 biopsies were taken from healthy skin without previous VZV or candida antigen challenge. Cells were counted within three to five most densely populated PV infiltrates, and the mean number of cells was plotted per PV. Error bars represent mean ± SEM. (**e, f**) Quantification of proliferating Ki67^+^ CD3^+^ T and Ki67^+^ CD20^+^ B cells within candida and VZV biopsy sections described in **a** and **b**. Error bars represent mean ± SEM. Data were analyzed with one-way ANOVA with Tukey’s multiple comparison post-test. (**g**) Representative immunofluorescence staining of proliferating (CD20^+^Ki67^+^, yellow cells, indicated by white arrows) B cells within day 7 skin biopsy sections (candida, top panel; VZV, bottom panel). Original magnification: ×20. Bar = 50 μm. ∗*P* < 0.05, ∗∗*P* < 0.01, ∗∗∗*P* < 0.001, ∗∗∗∗*P* < 0.0001. PV, perivascular; VZV, varicella-zoster virus.

In conclusion, this work highlights the recruitment and possible contributions of B cells to cutaneous immune responses in healthy human skin in a human in vivo system. Intradermal challenge with recall antigens induces in situ mature B-cell accumulation and proliferation, highlighting a previously undescribed B-cell component at sites of cutaneous antigen challenge. A potential model can therefore be envisaged in which B-cells traffic from the circulation to cutaneous sites of antigen re-exposure likely through the expression of skin-homing markers such as CLA and proliferate locally. However, the differentiation of B cells into antibody-secreting cells may not be involved in their function during antigen-specific responses in the skin. Further studies are required to identify the signals that control the migration of B cells to cutaneous sites and their functional capabilities in situ.

### Ethics statement

All human studies were approved by the National Research Ethics Service Committee London (United Kingdom) (“The Effects of Ageing on the Cutaneous Immune System,” Research Ethics Committee reference number: 11/LO/1846). Written informed consent was obtained from all volunteers before inclusion in this study.

### Data availability Statement

All of the data for the paper are included in the paper itself and within the figures; there is no additional dataset from which we have extrapolated data.

## ORCIDs

Isioma U. Egbuniwe: http://orcid.org/0000-0003-3752-3422

Robert J. Harris: http://orcid.org/0000-0003-4014-4589

Mano Nakamura: http://orcid.org/0000-0002-0699-6799

Frank O. Nestle: http://orcid.org/0000-0003-1033-5309

Arne N. Akbar: http://orcid.org/0000-0002-3763-9380

Sophia N. Karagiannis: http://orcid.org/0000-0002-4100-7810

Katie E. Lacy: http://orcid.org/0000-0001-9694-9197

## Conflict of Interest

The authors state no conflict of interest.

## Figures and Tables

**Supplementary Figure S1:**
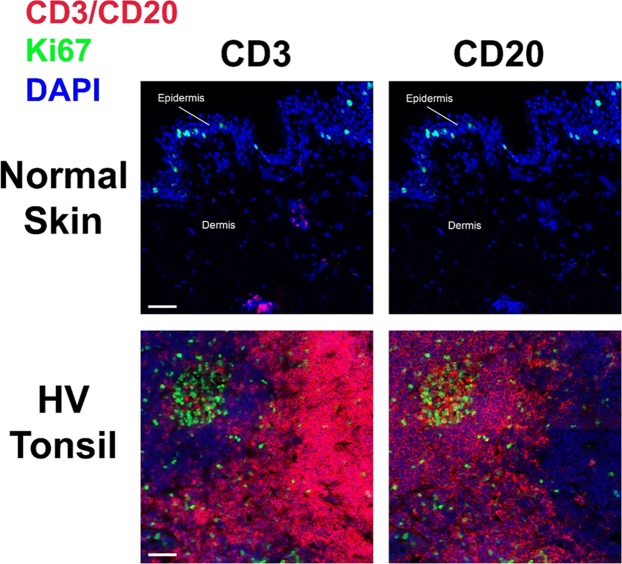
**B cells occur infrequently in normal unperturbed human skin.** Representative fluorescence immunohistochemistry images showing the relative absence of CD20^+^ B cells compared with that of CD3+ T cells within the dermis in normal human skin not challenged with VZV antigen (top panel). A large number of CD20^+^ B cells are present within tonsillar tissue (positive control, bottom panel). Original magnification: ×20. Bar = 50 μm. HV, healthy volunteer; VZV, varicella-zoster virus.

**Supplementary Figure S2:**
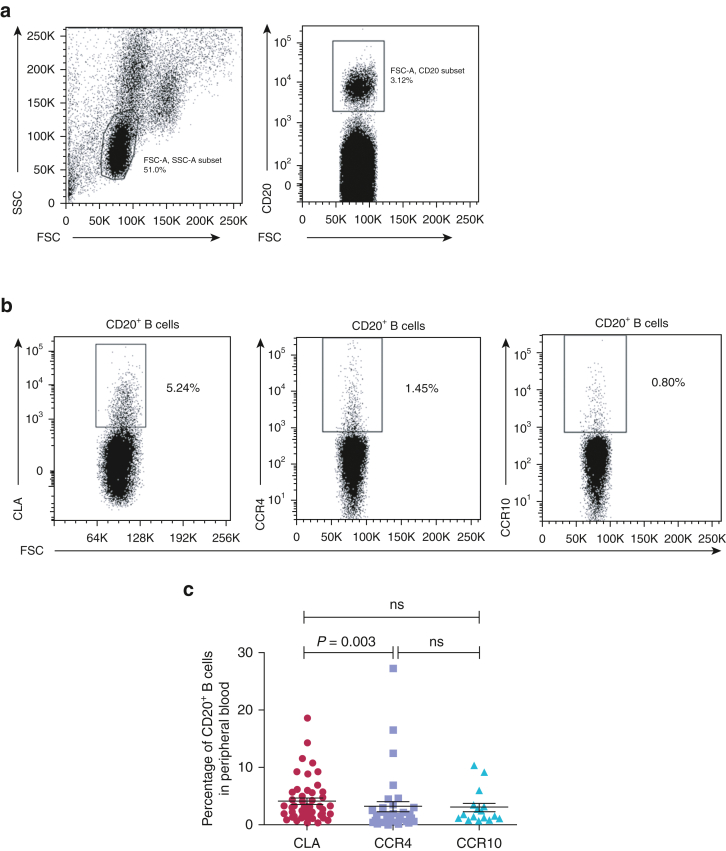
**Subsets of circulating B cells express skin-homing receptors CLA, CCR4, and CCR10 in healthy individuals.** Peripheral blood lymphocytes obtained from healthy donors were stained for flow cytometric analysis (FACSCanto II or LSRFortessa Cell Analyser - Becton Dickinson, Franklin Lakes, New Jersey) with antibodies specific for CD20 and the skin-homing markers CLA, CCR4, and CCR10. (**b**) The percentages of circulating CLA^+^CD20^+^ (left panel), CCR4^+^CD20^+^ (middle panel), and CCR10^+^CD20^+^ (right panel) were determined by successive gating on (**a**) the FSC versus on the SSC gate (left panel), followed by the CD20^+^ versus FSC gate (right panel). Flow cytometry plot is shown for one representative donor. (**c**) Scatter plots show the percentages of circulating CLA^+^CD20^+^ (n = 51), CCR4^+^CD20^+^ (n = 35), and CCR10^+^CD20^+^ (n = 16) B cells. Each symbol on the plot represents one individual. Error bars represent mean ± SEM. *P*-values were calculated using Kruskal‒Wallis test with Dunn’s multiple comparison post-test. CLA, cutaneous lymphocyte antigen; FSC, forward scatter; FSC-A, forward scatter area; K, thousand; ns, not significant; SSC, side scatter; SSC-A, side scatter area.

**Supplementary Figure S3:**
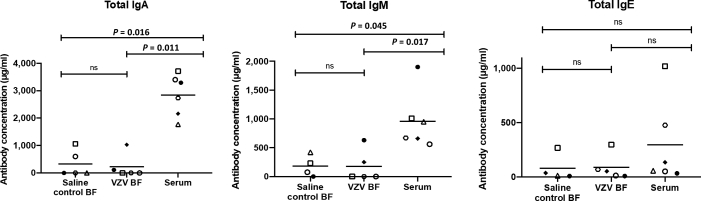
**Assessment of antibody titres in VZV and saline blister fluid compared with those in total donor serum.** Total IgA, IgM, and IgE antibody titres were analyzed within matched BF aspirates from saline control (n = 4) and VZV (n = 5) challenge sites as well as from donor sera (n = 6) on day 7 after VZV or saline challenge, using an ELISA-based technique. Each symbol on the plot represents one individual. Data were analyzed with Kruskal‒Wallis test with Dunn’s multiple comparison post-test. BF, blister fluid; ns, not significant; VZV, varicella-zoster virus.

**Supplementary Figure S4:**
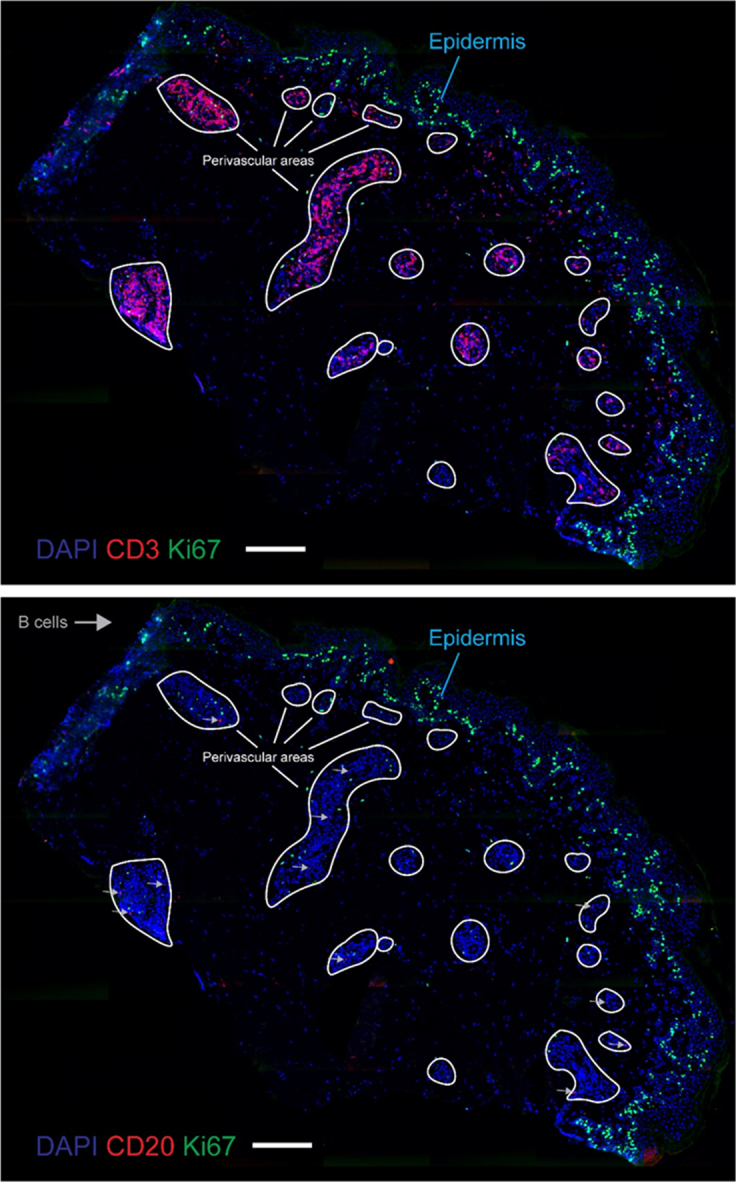
**Representative whole-slide images of day 3 VZV skin tissue showing the accumulation of CD3^+^ (top, red) and CD20^+^ (bottom, red) immune cells at perivascular sites (outlined in white).** Representative immunofluorescence whole-slide images of DAPI (blue), CD3 (red, top image), CD20 (red, bottom image), and Ki67 (green) staining. Where appropriate, CD20^+^ B cells are indicated with a gray arrow. White-outlined regions indicate perivascular areas. Bar = 200 μm.

## Supplementary Materials and Methods

### Varicella-zoster virus/candida skin testing

All human studies were approved by the National Research Ethics Service Committee London (United Kingdom) (“The Effects of Ageing on the Cutaneous Immune System,” Research Ethics Committee reference number: 11/LO/1846). Written informed consent was obtained from all volunteers before inclusion in this study. Healthy young adults (aged < 35 years; mean age = 26 years) were recruited for varicella-zoster virus (VZV; provided by Prof A.N. Akbar, University College London, UK) or candida skin testing (Allermed Laboratories, San Diego, CA) on the basis of previous exposure or active immunization. A total of 0.02 ml of VZV or Candin skin test antigen solution was administered intradermally into the anterior aspect of the forearm, which was confirmed by noticeable swelling at the injection site. As a control, 0.02 ml of sterile saline (Advanz Pharma, London, UK) solution was also injected into a corresponding area on the contralateral forearm. Punch biopsies of 5 mm were taken from sites of antigen testing 24 hours, 3 days, and 7 days after challenge for immunofluorescence analysis. Alternatively, suction blisters were induced over sites of antigen challenge as described below. Day 0 control biopsies were also taken from donors not subjected to previous VZV or candida antigen challenge.

### Induction of skin suction blisters

Skin suction blisters were induced at room temperature as previously described (Akbar et al., 2013). Briefly, 25–40 kPa negative pressure was applied for 2–3 hours by means of a suction chamber over sites of VZV and candida or saline intradermal injection either on day 3 or day 7 after the challenge. Suction pump pressure was gradually increased until a unilocular blister was formed, after which the pressure was gradually reduced to zero. Blisters were covered with protective dressing overnight, after which, blister fluid was aspirated and analyzed by flow cytometry and ELISA.

### FACS analysis

Single-cell lymphocyte suspensions were isolated by enzymatic digestion from surgically excised skin of patients undergoing breast reconstructive surgery at Guy’s and St. Thomas’ Hospitals, London, United Kingdom. Lymphocytes were also isolated from suction blister aspirates and healthy donor peripheral blood samples. Suspensions were stained with the following antibodies: CD3 FITC (clone SK7, BioLegend, San Diego, CA), CD19 V500 (clone HIB19, BD, Franklin Lakes, NJ), CD20 PerCpCy5.5 (clone L27, BD), CD22 PeCy7 (clone SJ10.1H11, Beckman Coulter, Brea, CA), CD27 V450 (clone M-T271, BD), and CLA PE (clone HECA-452, Miltenyi Biotec, Bergisch Gladbach, Germany). Isotype-matched antibodies or fluorescence minus one samples were run in parallel as controls. Samples were acquired on a FACSCanto II or LSRFortessa Cell Analyser (BD) using DIVA software.

### Immunofluorescence staining

Fresh frozen tissue sections were prepared from skin antigen‒challenged biopsy sections. Sections were blocked with goat serum and stained with primary antibodies against CD3 (mouse anti-human, clone F7.2.38, Dako, Santa Clara, CA), CD20 (mouse anti-human, clone L26, Dako), and Ki67 (rabbit anti-human, polyclonal, Abcam, Cambridge, United Kingdom). Corresponding isotype controls were also included. Sections were secondarily stained with Alexa Fluor 555 goat anti-mouse IgG or Alexa Fluor 488 goat anti-rabbit IgG antibodies. Cell nuclei were stained with DAPI (Invitrogen, Waltham, MA). Images were viewed and acquired using either a Zeiss Axiophot fluorescence microscope and AxioVision 4.8.1 software (Carl Zeiss, Oberkochen, Germany) or Olympus VS120-S5 for whole-slide imaging.

### Numerical evaluation of skin-infiltrating lymphocytes

Tissue sections singly stained for CD20 or CD3 (Alexa Fluor 555, red) and doubly stained for CD20/Ki67 or CD3/Ki67 (Alexa Fluor 555, red; Alexa Fluor 488, green; seen as yellow in double-positive cells) were counted to determine the number of infiltrating B (CD20+) and T (CD3+) lymphocytes as well as their proliferation status (according to Ki67 expression). Overall, 3–5 most densely populated areas of lymphocyte infiltration were selected per high power field per section, from which counts were taken of CD20^+^ (red), CD20^+^Ki67^+^ (yellow), CD3^+^ (red), and CD3^+^Ki67^+^ (yellow) cells. Average values were then derived for each lymphocyte group assessed. Only dermal perivascular infiltrating cells were included in counts. Whole-slide images (or ×10 magnification where appropriate) were initially captured to ensure a clear distinction was made between the epidermis and the dermis before cell counts were performed.

### ImmunoCAP assay

Total antibody titres in blister fluid and serum samples were assessed by Viapath (King’s College London, United Kingdom) using the ImmunoCAP methodology, which allows for accurate quantitation of antibodies of interest within human serum or plasma samples, regardless of antibody affinity, while also avoiding nonspecific binding. This is achieved by the use of a sandwich immunoassay with extremely high total binding capacity, combined with optimal amounts of cellulose in each solid phase. For example, to measure total serum IgE, an anti-IgE antibody is first covalently bound to the solid phase, to which serum IgE antibodies subsequently bind on the addition of the test sample. Unbound IgE antibodies are then washed off, after which, enzyme-labeled antibodies (directed against the bound IgE) are added to the reaction. After a final wash step, a developing agent is added, which binds to the anti-IgE‒IgE‒enzyme‒labeled antibody complex, giving off a fluorescence readout that is directly proportional to the concentration of the target antibody (IgE in this case) contained within the sample.

### Total IgG ELISA

Total IgG antibody titres in VZV- and candida-challenged skin blister fluid and healthy volunteer sera samples were also measured by sandwich ELISA. ELISA plates (VWR International, Radnor, PA) were coated with 100 μl per well of 10 μg/ml goat anti-human IgG capture antibody (SouthernBiotech, Birmingham, AL). Plates were sealed and incubated overnight, after which, wells were blocked for an hour using 100 μl of blocking buffer per well. Donor samples and IgG standards (8–0.00256 μg/ml; Sigma-Aldrich, St Louis, MO) were serially diluted in ELISA dilution buffer, and 50 μl per well were added in triplicate to ELISA plates. A total of 50 μl per well of horseradish peroxidase‒conjugated goat anti-human IgG (Merck, Darmstadt, Germany) secondary antibody was added, and detection was performed using OPD substrate (Thermo Fisher Scientific, Waltham, MA). The resulting data were analyzed using the Omega data analysis software (BMG Labtech, Ortenberg, Germany). Total IgG titres (mg/ml) were interpolated using the standard curve generated under a four-parameter logistic regression and were adjusted for dilution factor.

### Cell lysate‒based ELISA for the detection of VZV-specific antibodies

For the assessment of specificity of antibodies present with VZV- and candida-challenged skin blister fluid and healthy volunteer sera samples, we developed a cell lysate‒based ELISA assay. ELISA plates (VWR International) were coated with 100 μl per well of VZV lysate (Insight Biotechnology, Wembley, United Kingdom) diluted at 1:100 with PBS, in place of a capture antibody. Plates were sealed and incubated overnight, after which, wells were blocked for an hour using 100 μl of blocking buffer per well. Donor samples were serially diluted in ELISA dilution buffer, and 50 μl per well were added in triplicate to ELISA plates. A total of 50 μl per well of horseradish peroxidase‒conjugated goat anti-human IgG (Merck) secondary antibody was added, and detection was performed using OPD substrate (Thermo Fisher Scientific). The resulting data were analyzed using the Omega data analysis software (BMG Labtech). Antibody binding to VZV antigen was read out as optical density or relative absorbance. Briefly, the reactivity of specific antibodies to plated VZV lysate was evaluated as the mean optical density of blister fluid (VZV or candida) or donor sera (calculated from triplicate wells) at 1:100 dilution, relative to the mean optical density of the positive control anti-VZVgE antibody (16 ng/ml; Merck).

### Statistical analysis

All statistical analysis was performed using GraphPad Prism, version 5.03 (GraphPad Software, San Diego, CA). Collated data were initially assessed for normality of distribution using the D’Agostino and Pearson Omnibus normality test. In cases of small sample sizes (n ≤ 5), data were assumed to be non-normally distributed (nonparametric). No statistical analysis was performed for datasets with n ≤ 3.
